# Erythropoietin improves operant conditioning and stability of cognitive performance in mice

**DOI:** 10.1186/1741-7007-7-37

**Published:** 2009-07-08

**Authors:** Ahmed El-Kordi, Konstantin Radyushkin, Hannelore Ehrenreich

**Affiliations:** 1 Division of Clinical Neuroscience, Max Planck Institute of Experimental Medicine, Göttingen, Germany

## Abstract

**Background:**

Executive functions, learning and attention are imperative facets of cognitive performance, affected in many neuropsychiatric disorders. Recently, we have shown that recombinant human erythropoietin improves cognitive functions in patients with chronic schizophrenia, and that it leads in healthy mice to enhanced hippocampal long-term potentiation, an electrophysiological correlate of learning and memory. To create an experimental basis for further mechanistic insight into erythropoietin-modulated cognitive processes, we employed the Five Choice Serial Reaction Time Task. This procedure allows the study of the effects of erythropoietin on discrete processes of learning and attention in a sequential fashion.

**Results:**

Male mice were treated for 3 weeks with erythropoietin (5,000 IU/kg) versus placebo intraperitoneally every other day, beginning at postnatal day 28. After termination of treatment, mice were started on the Five Choice Serial Reaction Time Task, with daily training and testing extending to about 3 months.

Overall, a significantly higher proportion of erythropoietin-treated mice finished the task, that is, reached the criteria of adequately reacting to a 1.0 sec flash light out of five arbitrarily appearing choices. During acquisition of this capability, that is, over almost all sequential training phases, learning readouts (magazine training, operant and discriminant learning, stability of performance) were superior in erythropoietin-treated versus control mice.

**Conclusion:**

Early erythropoietin treatment leads to lasting improvement of cognitive performance in healthy mice. This finding should be exploited in novel treatment strategies for brain diseases.

## Background

The haematopoietic growth factor erythropoietin (EPO) has been in clinical use for over 20 years to treat patients with anaemic conditions, ranging from renal failure to cancer. Upon introduction of EPO to the clinic, it was observed that cognitive performance of treated individuals also improved. This improvement was essentially attributed to anaemia correction with subsequently enhanced tissue oxygenation also in the brain [1]. Much later, EPO and its receptor were found to be produced by and act on cells of the nervous system [2,3]. Many reports on neuroprotective and neuroregenerative effects of EPO in rodent models of neurological diseases followed [4] (for review see [5,6]). Our recent human studies in schizophrenia and multiple sclerosis revealed profound EPO effects on cognitive performance [7,8]. The separation of haematopoietic and neuroprotective properties upon slight modification of the EPO molecule ultimately proved the haematopoiesis-independent effect of EPO on the nervous system [6].

To investigate, in the absence of interfering disease variables, the physiological role of the brain EPO system regarding cognition, we performed a series of studies in healthy young mice where we found hippocampal memory, measured by classical fear conditioning, improved after 3 weeks of EPO treatment. At the time of improved memory, an increase in short-term and long-term potentiation was documented in hippocampal slices [9]. Along the same lines, application of a single high intravenous dose of EPO in healthy human volunteers enhanced the hippocampal response during memory retrieval measured by functional magnetic resonance imaging one week later [10]. It should, however, be also mentioned that preclinical studies using disease models failed to show effects of EPO treatment on cognition in their respective healthy control groups (for example, [11,12]). This is most likely explained by the application of tests (for example, T-maze, 8-arm radial maze), well suited to measure pathology but less sensitive for assessing subtle improvements in cognitive performance in healthy individuals.

Since the development of new therapies, targeting cognitive performance is of major interest for clinical neuroscience, and EPO might here be a promising candidate; thus the spectrum of cognitive functions potentially influenced by EPO has to be better defined. For this purpose, we employed the Five Choice Serial Reaction Time Task (5CSRTT), originally developed for rats by Carli and colleagues [13] and adapted for mice by Humby and co-workers [14]. The 5CSRTT has been used to analyse attention and executive functions in different rodent models of neuropsychiatric diseases, including attention-deficit/hyperactivity disorder [15], schizophrenia [16] and impulsivity disorders [17] (for review, see [18]). With 5CSRTT, we systematically studied the effect of EPO on different types of learning, memory and attention in healthy young mice. We note that obtaining cognitive results in healthy young individuals cannot automatically be translated to disease situations or aged mice. Nevertheless, this approach is the simplest first step to providing a foundation for mechanistic insight. We report here that early EPO treatment indeed improves most of the sequential learning and memory components of a complex long-term cognitive task, ultimately leading to better and more stable cognitive achievements.

## Results

### Erythropoietin increases overall performance in 5CSRTT

**Figure 1:**
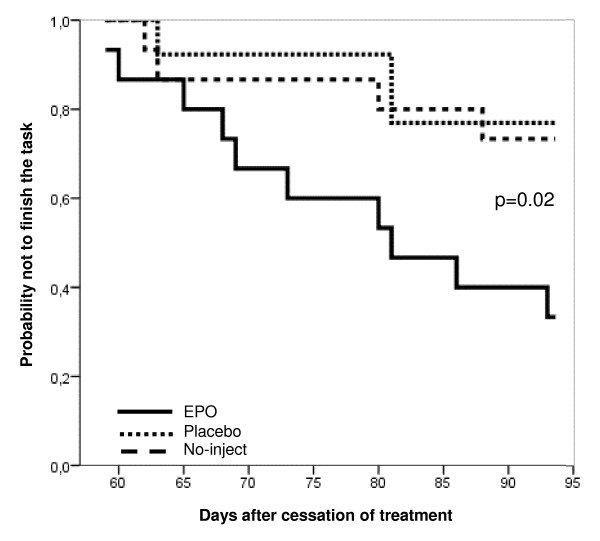
**Kaplan-Meier presentation of overall group performance in mice**. Curves represent group performance (*n *= 13–15 per group). They indicate the cumulative probability of members of each group not to finish the task (that is, not to reach performance criteria in the 1.0-sec stimulus duration phase). EPO-treated mice are superior, that is, have a lower probability not to finish the task as compared with no-inject and placebo groups. On day 59 after cessation of treatment, the first mouse reached criteria in the 1.0-sec phase. This mouse belonged to the EPO group. Day 94 is the time point on which > 60% of one experimental group (here the EPO group) had finished the task.

First, we compared the overall performance of EPO, placebo, or no-inject groups in 5CSRTT. Kaplan-Meier analysis revealed that the proportion of mice that finished training in the 5CSRTT (that is, reached criteria of stably responding to a 1.0-sec stimulus duration on three consecutive days) up to day 94 after cessation of treatment was significantly higher in the EPO-treated group as compared with placebo and no-inject groups (Log-Rank test, *P *= 0.02). There were no differences between placebo and no-inject groups (Figure 1). Taken as a group, EPO-treated mice learn faster.

### Erythropoietin accelerates associative, operant and discriminant learning in 5CSRTT

**Figure 2:**
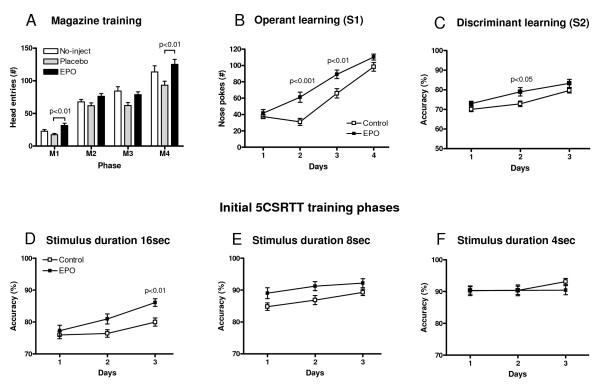
**Analysis of distinct sequential learning phases**. **(A) **Number of head entries as indicators of associative learning in magazine training (M1–4) show an overall significant effect of EPO treatment compared with placebo. **(B) **Number of nose pokes in the operant learning phase (S1) as well as **(C) **percentage of correct nose pokes in the discriminant learning phase (S2) were higher in the EPO-treated compared with the control group (placebo plus no-inject). **(D, E) **Initial cognitive performance in the 5CSRTT was better upon EPO treatment in the 16-sec and 8-sec stimulus duration phases, but no longer in the 4-sec phase **(F)**; *n *= 14–28 per group; data presented as mean ± S.E.M.; all significance values refer to *post hoc *tests which were only significant if ANOVA was also significant; in **(E)**, only ANOVA was significant (*P *= 0.03).

Since different types of learning determine performance in the 5CSRTT, we asked whether the superiority of EPO-treated mice is reflected in initial learning parameters. Indeed, the number of head entries during habituation and magazine training appeared higher throughout all phases (M1–M4) in EPO-treated mice compared with the placebo group (Figure 2A). A significant difference between groups in phases M1 and M4 of magazine training by Kruskal-Wallis test (*P *< 0.05 for each) could be attributed to superior performance of the EPO versus placebo group following *post hoc *analysis (Dunn's multiple comparison test, *P *< 0.01). During the operant learning phase (S1), EPO-treated mice made more nose pokes (ANOVA: *F*_(1,41) _= 8.81, *P *= 0.005) on days 2 and 3 (*post hoc P *< 0.001 and *P *< 0.01, respectively) (Figure 2B). Additionally, there was a significant group effect in the discriminant learning phase (S2), with EPO-treated mice demonstrating higher choice accuracy (ANOVA: *F*_(1,41) _= 5.35, *P *= 0.026) (Figure 2C).

We wondered whether the improvement in operant and discriminant learning parameters would continue in face of more complex conditions and thus enhanced cognitive challenge during the 5CSRTT training. In fact, the 5CSRTT procedure is much more complex than the preceding shaping phases and consists of more parallel stimuli in addition to stimulus duration (for example, number of stimulus lights, more restricted time-out period). We analysed accuracy in the consecutive stimulus duration phases: 16 sec, 8 sec and 4 sec. The percentage of correct responses over the first 3 days of each phase served as readout of initial learning capabilities in the 5CSRTT training (Figure 2D, E and 2F). EPO-treated mice performed better in 16-sec and 8-sec phases (ANOVA *F*_(1,41) _= 7.21; *P *= 0.01 and *F*_(1,39) _= 4.87; *P *= 0.03, respectively, Figure 2D and 2E), but no longer in the following ones, from 4 sec (Figure 2F) to 2 sec, 1.8 sec, 1.4 sec, 1.2 sec and 1.0 sec (data not shown). Overall accuracy increased over consecutive phases until reaching a plateau at 4 sec for both groups, which then stayed essentially stable over the following shorter-duration phases (data not shown). There was no consistent difference in accuracy (nor amount of omissions) during the attentional challenge phase of 0.8 sec (*F*_(1,18) _= 0.49, *P *= 0.5 and *F*_(1,18) _= 1.45, *P *= 0.24, respectively).

### Erythropoietin improves task adaptation and stabilizes performance in 5CSRTT

**Figure 3:**
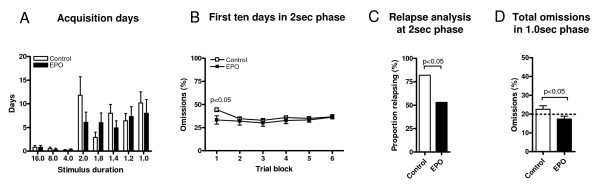
**Analysis of selected readouts of cognitive performance in high-performer mice**. **(A) **Number of acquisition days required for reaching performance criteria at each stimulus duration phase shows no group differences but a sharp increase in the transition from 4 sec to 2 sec. **(B) **The EPO-treated group showed faster task adaptation: The proportion of omissions in the first of six 10-trial blocks collapsed across the first 10 days of the 2-sec phase was significantly lower. *Post hoc *test. **(C) **The EPO-treated group showed higher stability of performance: the proportion of mice that relapsed from already reached performance criteria in the 2-sec stimulus duration phase was smaller. Chi^2 ^test. **(D) **EPO improves performance stability even in the 1-sec phase: Proportion of total omissions over 4 days after reaching performance criteria was significantly lower in the EPO group. Mann-Whitney test. *N *= 7–28; data presented as mean ± S.E.M.

We next analysed the number of training days required to reach performance criteria in 5CSRTT for each stimulus duration, that is, acquisition days (Figure 3A). There was a significant effect of stimulus duration on the number of training days needed, independent of treatment (*F*_(7,126) _= 8.24, *P *< 0.0001). Starting immediately from 2 sec, mice required more days to reach criteria compared with previous phases. To further address the phenomenon of an abrupt increase of training days in the 2-sec phase (independently of treatment), we analysed omissions more closely. In fact, omissions in all training phases from 16 sec to 1 sec were comparable between the two experimental groups, and rates were low up to the switch from the 4-sec to the 2-sec phase (for illustration, data of the respective first training day of each stimulus duration are given as an example: range of omissions on day 1 of 16 sec: 6.55 ± 4.28 EPO versus 5.83 ± 4.17 control; on day 1 of 8 sec: 9.45 ± 13.09 EPO versus 8.93 ± 8.99 control; on day 1 of 4 sec: 16.90 ± 9.94 EPO versus 16.90 ± 9.81 control; on day 1 of 2 sec: 29.78 ± 12.86 EPO versus 35.89 ± 10.27 control; on day 1 of 1.8 sec 22.11 ± 7.46 EPO versus 21.96 ± 8.75 control; on day 1 of 1.4 sec: 27.00 ± 7.58 EPO versus 28.93 ± 11.12 control; on day 1 of 1.2 sec: 24.67 ± 8.21 EPO versus 24.1 ± 8.77 control; on day 1 of 1.0 sec: 24.7 ± 5.72 EPO versus 29.06 ± 10.29 control). It turned out that from the 4-sec phase to the 2-sec phase, omissions promptly doubled and stayed at a high level up to the end of 5CSRTT training (1 sec), independent of treatment group. This may explain the prominent increase in training day requirement starting from 2 sec.

We next averaged omitted trials in each of the six 10-trial blocks over the first 10 days of this 2-sec phase (Figure 3B). There was no significant overall effect of treatment; however, there was a significant interaction between trial block and treatment (*F*_(5,130) _= 2.37, *P *= 0.04). *Post hoc *analysis revealed that EPO-treated mice had significantly fewer omissions in the first trial block compared with controls (*P *< 0.05), pointing to a faster adaptation to the task.

As fluctuations in performance may increase the number of days needed for reaching performance criteria, we analysed the magnitude of fluctuation ('relapsing back from already achieved criteria') in the 2-sec phase. Here, the control group had significantly more relapses compared with the EPO group, pointing to more stable performance upon EPO (Chi^2 ^test, *P *= 0.04) (Figure 3C). To further explore the effect of EPO on stability of performance, we analysed total omissions after reaching criteria (1.0 sec) over additional 4 days. EPO-treated mice were superior (*P *= 0.04) as compared with the control group (Figure 3D).

### Erythropoietin does not affect locomotor activity in 5CSRTT

To clarify whether increased locomotion has contributed to the superior performance of EPO-treated mice in phases with 16-sec and 8-sec stimulus duration, we analysed the average of reward latency (that is, the time between responding correctly and collecting reward) as well as the 'latency correct' (that is, the time between stimulus presentation and responding correctly) in the corresponding phases. There were no differences between groups in either reward latency or in 'latency correct' in any phase of the 5CSRTT training (data not shown).

## Discussion

In the present study, a 3-week high-dose EPO treatment of healthy young mice increased the probability of these animals finishing training in the 5CSRTT. While there were no differences in the number of total days needed to terminate the task among ultimately successful individual mice, regardless of group assignment, the proportion per group was different, with more successful mice in the EPO-treated cohort. EPO-treated mice showed superior performance in associative, operant and discriminant learning as well as in initial 5CSRTT training phases. Moreover, EPO-treated mice demonstrated better task adaptation and higher performance stability. In contrast, with the number of mice remaining in the task, there were no clear effects with this particular EPO treatment schedule (terminated more than 3 months before) on attentional performance, as defined by response to the 0.8-sec stimulus duration.

To gain an overall impression of the progress in cognitive training of our mouse groups, we employed the survival analysis of Kaplan-Meier. This methodological approach was crucial for our purposes as it describes, over a long testing period, the development of group performance in a higher cognitive task, the 5CSRTT. By applying standard statistical methods only, the clear superiority of the EPO group would not have been detectable. In fact, the Kaplan-Meier results inspired us to dissect out potential learning processes contributing to the superior group performance of EPO-treated mice. The success of this statistical approach would suggest the usefulness of this method for future group analysis in the 5CSRTT.

While clear benefits of EPO treatment could easily be demonstrated throughout all initial learning phases including the 5CSRTT training up to 8-sec stimulus duration, we did not find differences in accuracy in phases with stimulus durations of 4 sec and below. This might be due to a ceiling effect, as both groups had reached almost maximal performance at this stage. Nevertheless, also in these more progressed phases, EPO superiority was visible when analysing more subtle readouts of cognitive performance, for example, stability and task adaptation.

A particularly difficult 5CSRTT training step for mice is the switching from the 4-sec to the 2-sec stimulus duration phase. In this challenging phase, EPO-treated mice had consistently lower omission rates when entering the task, indicating immediate task adaptation. This improvement just failed to translate into significant differences in acquisition days. Interestingly, a previous study on galanin transgenic mice, a model for impaired learning capacity, reported for this critical 2-sec phase more than doubling of required training days compared with controls, due to more omissions [19]. In contrast to the improved task adaptation shown in the present study, reflected by less omissions in the first trial block, galanin transgenic mice exhibited more omissions in the last two trial blocks, pointing to deficiency of sustained attention [19]. In contrast to all other studies employing 5CSRTT, we have additionally analysed parameters representing stability of performance. The analysis of relapse frequency (that is, dropping down from a once-acquired performance level) in the challenging 2-sec stimulus duration phase with its increasing attentional challenge again revealed a more consistent performance by the EPO group.

An important limitation of the current study (and of 5CSRTT in general) has to be addressed: the strong selection bias of mice with higher cognitive abilities means that mice that do not learn crucial steps of the task are excluded from further testing (which is frequently not mentioned in respective publications). This gradual decrease of numbers in advanced training phases renders statistical analysis increasingly difficult. For instance, in the current study, there would not have been enough mice in the placebo and the no-inject group for separate analysis in the attentional phase with stimulus duration of 0.8 sec, despite starting out with 15 mice per group. A total of 45 mice to be run simultaneously in this task already reaches the limits of a generous set-up of five chambers and more than a full working day of the investigator. Since performance of placebo and no-inject groups did not differ much in the early phases, pooling of both groups to obtain a 'control cohort' was possible.

## Conclusion

The findings of the present study, that is, improved sequential learning and memory components of a complex long-term cognitive task upon EPO treatment, will provide the basis for further work targeting molecular facets of these critical phases. Such studies will include quantitative and qualitative evaluation of neurogenesis and synapse formation in respective brain regions, for example, cingulate cortex and hippocampus. In the latter, we recently detected increased long-term potentiation after EPO treatment [9]. In addition, future work will have to assess whether the effects of EPO obtained here are restricted to the use of very young and healthy animals, or would be similarly strong in older mice and/or disease conditions. Further untangling of molecular mechanisms of EPO action on higher cognitive functions may then ultimately open new avenues for prevention strategies and therapeutic interventions in neuropsychiatric diseases.

## Methods

### Animals

Forty-five male C57BL/6NCrl mice (Charles River, Sulzfeld, Germany), 3 weeks old upon arrival, were housed in groups of five in standard plastic cages, with food and water *ad libitum*. The temperature in the colony room was maintained at 20–22°C, the light-dark cycle at 12 h (light on at 0400 hrs). After 7 days of acclimatising to the new environment, injections were started at the age of 28 days and were always performed in the first half of the light phase. Behavioural experiments were conducted by an investigator, blinded to treatment condition, during the second half of the light phase (between 1000 hrs and 1500 hrs). All experiments were approved by the local Animal Care and Use Committee in accordance with the German Animal Protection Law.

### Injection protocol

Mice were randomly assigned to one of three groups: EPO, placebo or 'no-inject' (to uncover potential effects of repeated injection stress), each consisting of 15 mice. Mice were intraperitoneally injected every other day for 21 days (11 injections in total) either with EPO (5,000 IU/kg, Epoetin-alpha, Janssen-Cilag, Neuss, Germany) or with placebo (diluent buffer) in a volume of 0.01 ml/g body weight. Mice from the no-inject group were just weighed every other day (in order to keep handling similar to the injected mice) with no additional manipulations. Training started 1 day after cessation of injections.

### 5CSRTT apparatus

Mice were trained in an operant chamber (height 18 cm, width 15.5 cm, depth 20 cm, Med Associates Inc, St. Albans, USA), enclosed in a sound-attenuating box and connected to a Fujitsu Siemens PC. One wall of the operant chamber had a curved shape and carried an array of five stimulus holes. The stimulus holes were 1.2 cm in diameter and contained an LED stimulus light (depth 1 cm) in the rear. Infrared photocell pairs were located at 4 mm from the entrance of the stimulus holes and detected nose pokes of mice into the holes. The wall opposite to the stimulus holes contained a magazine cup, also with a photocell detector of head entries, in which liquid reward (4% sucrose solution) was delivered always simultaneously with illumination of the magazine. The house light was located 32 cm above the magazine.

### Habituation and magazine training

**Figure 4:**
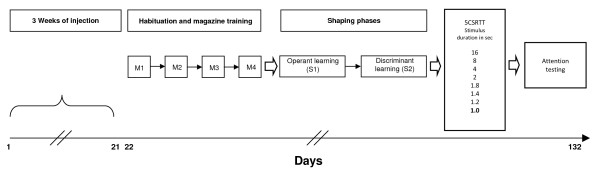
**Experimental design**. Following 3 weeks of EPO (5,000 IU/kg body weight intraperitoneal) versus placebo (diluent control) treatment or handling only (no-inject group), 7-week-old mice go through habituation/magazine training (M1–M4) and shaping phases (S1–S2) before starting training in the 5CSRTT. Here they move from 16 sec to the respective next phase with lower stimulus duration (8 sec and so on) upon reaching fixed criteria. Having reached criteria in the 1.0-sec phase, their attention is challenged by further shortening stimulus duration (0.8 sec).

Two days before starting training, mice were habituated to the liquid reward of 4% sucrose solution in their home cages overnight. The day before starting magazine training, sucrose bottles were removed and mice were water-deprived. Water deprivation was applied during the whole experimental period. Immediately after finishing the daily test sessions, mice were given water in individual cages for 20 min. Magazine training consisted of four consecutive phases (M1–M4), one phase per day, each lasting for 15 min, with all stimulus holes closed. In the first phase (M1), liquid reward was delivered (10 μl) upon initiation of the training session. In the second phase of magazine training (M2), the number of potential rewards was increased, with a fixed interval of 118 sec between reward presentations. A head entry into the magazine was required to collect the reward. In the third phase (M3), the fixed interval was replaced by a head entry-dependent interval of 100 sec to obtain reward. In the last phase (M4), this interval was further reduced to 50 sec, ideally yielding a consistently increasing number of head entries. Head entries into the magazine together with reward consumption were taken as indicators for associating the magazine with reward delivery (see experimental design Figure 4).

### Shaping phases (operant and discriminant learning)

During shaping, mice were trained to perform a nose poke into an illuminated stimulus hole in order to obtain reward. The shaping procedure consisted of two phases (each extending over several days, dependent on individual performance, and with a daily session duration of 30 min) where mice were taught to associate nose poking into an illuminated hole with reward (phase S1), and then trained to discriminate between those nose pokes that lead to reward (illuminated holes) and those that do not (unlit holes) (phase S2) (Figure 4). Throughout shaping all stimulus holes were open. During S1, all stimulus lights were on. Any nose poke in a stimulus hole was rewarded. The inter-trial interval (time from pick-up of reward to next stimulus hole illumination) was set to 8 sec. During S2, presentation of lit and unlit holes was conducted in a pseudorandom manner. Mice were only rewarded upon nose poking into a lit stimulus hole. Performing a nose poke in an unlit hole led to switch-off of the house light for 5 sec. Mice in S1 were moved to the next training phase once they had reached 35–40 nose pokes each on three consecutive days. S2 was terminated when mice had arrived at a stable performance of ≥ 70% correct responses. Starting from analysis of shaping phases, placebo and no-inject groups were pooled, since they no longer differed in any of the high-performing tasks beyond habituation.

### 5CSRTT training

The training session started with illumination of magazine light and presentation of 4% sucrose solution. Head entry started the trial. At 8 sec after head entry, light (initially set to 16 sec) was randomly presented in one of the five stimulus holes. A correct response, that is, nose poking into the lit hole, led to reward (6 μl) and next trial start after 8 sec. Nose poking in an unlit stimulus hole, that is, an incorrect response, led to extinguishing the house light for 5 sec (time-out) and no reward. Further nose pokes during time-out extended that period for additional 5 sec each. If a mouse did not respond by nose poking into any of the holes during stimulus presentation, an omission was counted. As a consequence, no reward was presented. Also omissions provoked time-out. A training session was terminated after 30 min or upon performing 60 trials, whichever came first. Mice were trained in the phase with 16-sec stimulus duration until they reached clearly defined performance criteria (≥ 75% accuracy (correct responses/correct + incorrect responses * 100), ≤ 20% omissions and at least 50 trials performed over three consecutive days). Eight such phases followed with gradually declining stimulus duration up to 1.0 sec (16, 8, 4, 2, 1.8, 1.4, 1.2, and 1.0 sec).

In the first phases, mice had time to respond as long as the stimulus light was on. For phases with stimulus duration below 5 sec, the response time (so-called limited hold) was added up to 5 sec. Having 'finished the task' meant having reached the above performance criteria for the 1.0 sec phase. Training sessions were performed every day, including weekends. As soon as > 60% of the mice in one of the three experimental groups had finished the task by reaching performance criteria in the 1.0 sec phase, regular 5CSRTT training was stopped for all groups (day 94; Figure 1) and only high performers of all groups were carried on with attention testing, that is, stimulus duration below 1.0 sec (0.8 sec). This resulted in EPO *n *= 10 versus control *n *= 10 (placebo *n *= 4 plus no-inject *n *= 6).

### Overview on parameters of task acquisition

The following parameters were employed: (1) proportion of mice that finished the task in all experimental groups; (2) number of days until reaching performance criteria in all phases (acquisition days); (3) proportion of omissions, that is, number of omitted per total number of performed trials within one training session; (4) number of head entries during a magazine training session; (5) number of nose pokes during an operant learning session; (6) accuracy, that is, percentage of correct responses calculated as number of correct responses/correct + incorrect responses*100; (7) sustained attention, that is, attentional performance, expressed as omissions as a function of time in session, evaluated over the first 10 days in the 2-sec phase (the most challenging training step). Data are expressed in 10-trial blocks collapsed across all 10 days; (8) newly introduced parameter: *Stability of performance/relapses*, that is, the ability to maintain a high performance with respect to omissions and accuracy for three consecutive days. Mice that reached the above mentioned criteria without keeping them for the required 3 days were considered relapsing. An additional measure of performance stability in high performers were omissions in the 1.0-sec stimulus duration phase, as determined for 4 days after reaching criteria.

### Statistical analysis

Statistical analysis was performed using the statistical programs SPSS for windows, release 16 (SPSS Inc., Chicago, USA) and GraphPad Prism version 4.00 for Windows, (GraphPad Software, San Diego, USA). We applied two-way ANOVA repeated measures, Kruskal-Wallis test, chi^2^-test and Mann-Whitney test where indicated. Bonferroni and Dunn's multiple comparison tests were used for *post hoc *analysis. Threshold for significance was *P *< 0.05. Survival analysis/Kaplan-Meier curves [20] were introduced to demonstrate the proportion of mice per group finishing the 5CSRTT training up to the 1.0-sec stimulus duration (task goal). Mice that did not reach the goal until day 94 were censored.

## Authors' contributions

AEK and KR carried out the behavioural experiments. AEK performed statistical analysis of experimental results. KR participated in the design of the study and in writing of the manuscript. AEK and HE wrote the manuscript. HE supervised the whole project and designed the study. All authors read and approved the final manuscript.
